# Identifying Protein Complexes from Dynamic Temporal Interval Protein-Protein Interaction Networks

**DOI:** 10.1155/2019/3726721

**Published:** 2019-08-21

**Authors:** Jinxiong Zhang, Cheng Zhong, Hai Xiang Lin, Mian Wang

**Affiliations:** ^1^School of Computer Science and Engineering, South China University of Technology, Guangzhou 510006, China; ^2^School of Computer, Electronics and Information, Guangxi University, Nanning 530004, China; ^3^Faculty of Electrical Engineering, Mathematics and Computer Science, Delft University of Technology, Delft 2628 XE, Netherlands; ^4^School of Life Science of Technology, Guangxi University, Nanning 530004, China

## Abstract

Identification of protein complex is very important for revealing the underlying mechanism of biological processes. Many computational methods have been developed to identify protein complexes from static protein-protein interaction (PPI) networks. Recently, researchers are considering the dynamics of protein-protein interactions. Dynamic PPI networks are closer to reality in the cell system. It is expected that more protein complexes can be accurately identified from dynamic PPI networks. In this paper, we use the undulating degree above the base level of gene expression instead of the gene expression level to construct dynamic temporal PPI networks. Further we convert dynamic temporal PPI networks into dynamic Temporal Interval Protein Interaction Networks (TI-PINs) and propose a novel method to accurately identify more protein complexes from the constructed TI-PINs. Owing to preserving continuous interactions within temporal interval, the constructed TI-PINs contain more dynamical information for accurately identifying more protein complexes. Our proposed identification method uses multisource biological data to judge whether the joint colocalization condition, the joint coexpression condition, and the expanding cluster condition are satisfied; this is to ensure that the identified protein complexes have the features of colocalization, coexpression, and functional homogeneity. The experimental results on yeast data sets demonstrated that using the constructed TI-PINs can obtain better identification of protein complexes than five existing dynamic PPI networks, and our proposed identification method can find more protein complexes accurately than four other methods.

## 1. Introduction

The majority of proteins interact with each other to perform a specific biological process [1]. The fast accumulation of protein-protein interaction (PPI) data has made maps of PPI networks of several model organisms become available [2]. Identifying protein complexes from PPI networks plays a key role in understanding cellular organizations and functional mechanisms [3].

Over the past decades, the studies on identifying protein complexes from static protein-protein interaction network (SPIN) have yielded many effective methods. The clustering-based methods such as MCODE [4], ClusterONE [5], MCL [6], PCP [7], APcluster [8], SPICi [9], and DPCLus [10] identify complexes by detecting closely connected structures from SPIN. Gavin et al. [1] discovered the core-attachment structure of yeast protein complexes based on genome-wide analysis. Accordingly, CORE [11], COACH [12], WPNCA [13], and MCL-CAw [14] were designed to find protein complexes from SPIN. Some methods [15–17] detect protein complexes with biological significance by integrating GO-based functional annotations and SPIN, and other methods [18, 19] measure Pearson correlation coefficient between two proteins and weight SPIN to identify protein complexes.

The aforementioned methods mainly focus on identifying complexes from static protein-protein interaction network (SPIN). However, the real PPI network in cell keeps changing over different stages of the cell cycle [20]. In fact, cellular systems are highly dynamic and responsive to environment cues [21]. So it is expected that modelling the real PPI network as dynamic PPI networks can lead to identifying more protein complexes accurately.

Fortunately, by monitoring simultaneous and quantitative changes in RNA concentration of thousands of genes under various experimental conditions, DNA microarray technology produced a large number of gene expression data [22, 23]. These gene expression data provide potential insights into the dynamics of PPI networks. Thus, the key step of identifying protein complexes from dynamic PPI networks is how to construct nearly real PPI networks using gene expression data. During a whole cell cycle, protein is not always active all the time. To construct the dynamic PPI network, it requires determining the so-called active time point at which protein exhibits activity. According to the periodicity of gene expression, De Lichtenberg et al. [24] constructed dynamic PPI networks over the yeast mitotic cell cycle by determining active time points of each protein. A protein is considered to be active when its level of gene expression exceeds a specified threshold. Tang et al. [22] used a recommended threshold to filter nonactive protein over three successive metabolic cycles and then constructed a time-course protein interaction network (TC-PIN). Instead of using a global threshold, Wang et al. [23] presented a three-sigma method, which uses the sum of the gene expression mean and three standard deviations as a threshold, to determine active time points of each protein, and constructed dynamic protein interaction networks (DPIN) and identified complexes from DPIN. Some swarm intelligence-based methods [25–29] also exploit the three-sigma method to construct dynamic PPI networks and identify protein complexes. Based on the three-sigma method, Zhang et al. [30] calculated the active probability of each protein at different time point to determine the active time point of each protein and constructed dynamic probabilistic protein interaction networks (DPPN). Furthermore, OU-Yang et al. [31] proposed a time smooth overlapping complexes detection (TS-OCD) model to construct dynamic PPI networks to detect temporal protein complexes. Shen et al. [32] used the deviation degree method to construct a Time-Evolving PIN (TEPIN) to detect temporal protein complexes. By adopting a dynamic model-based method to filter the noisy data from gene expression profiles, Xiao et al. [21] proposed a* k*-sigma method to determine whether a protein at a time point is active and constructed a noise-filtered active protein interaction network (NF-APIN) to detect protein complexes. The fore-mentioned methods mainly consider how to construct the dynamic PPI networks and then apply existing identification method to find protein complexes from the constructed dynamic PPI networks.

Furthermore, some researchers have not only investigated how to construct the dynamic PPI networks but also designed identifying methods to find protein complexes from the constructed dynamic PPI networks. By combining the active probability of proteins and Pearson correlation coefficient of PPIs with static PPI networks, Zhang et al. [33] constructed dynamic PPI networks and proposed a protein complex prediction method. Based on the neighbor affinity and dynamic protein-protein interaction network, DPC-NADPIN method [34] selects the proteins with a high clustering coefficient and their neighbors to consolidate into an initial cluster and iteratively expands the neighbor proteins to the cluster to form a protein complex. TS-OCD method [31] captures the temporal feature of networks between consecutive time points and detects temporal protein complexes from the constructed dynamic PPI networks. Shen et al. [35] proposed a method called DCA (Dynamic Core-Attachment), which uses three-sigma method to construct dynamic PPI network integrating the inherent organizations of protein complexes and applies an outward expanding strategy to identify protein complexes with the characteristic of core-attachment structure. All four above-mentioned works identify protein complexes by constructing dynamic PPI networks using gene expression data and topological features of PPI networks.

We observed that all the existing methods determine active time point of proteins by a conservative and relative high threshold. This leads to losing dynamic information of gene with expression value that is lower than the specified threshold. In this paper, we first exploited the undulating degree above the base level of gene expression instead of the gene expression level to determine the active time point of a protein and constructed temporal PPI networks (TPNs) by protein interaction data and gene expression data. We then proposed a method which not only converts TPNs into temporal interval PPI networks (TI-PINs) but also identifies more colocalized, coexpressed, and functionally significant protein complexes from the constructed TI-PINs by multisource biological data. Finally, we evaluated our constructed TI-PINs and other dynamic PPI networks and compared our proposed identification method with four other competing methods.

## 2. Methods

In this section, we describe how to construct temporal interval PPI networks (TI-PINs) and identify protein complexes from TI-PINs.

### 2.1. Preliminary

Let a graph* G*=(*V*,* E*) represent a static protein-protein interaction network (SPIN), where* V* is a set of nodes (proteins) and* N*=|*V*|,* E* is a set of edges (protein-protein interactions), and* e*(*i*,* j*) denotes the edge between nodes* i* and* j*, where* i*,* j*=1, 2,…,* N*. Let *S*_*ppi*_=(*PP*,* s*) denote a set of protein-protein interactions with reliability score, where* PP* is a set of interacting protein pairs and* s*(*x*,*y*) denote the reliability score of the interacting protein pair (*x*,*y*) in* PP*. Thus, we use* GW*=(*V*,* E*,* w*) to denote the graph* G *weighted by *S*_*ppi*_, where the edge weight* w*(*i*,* j*) is defined as follows:

(1) wi,j=si,j,if i,j∈PP,  ei,j∈E1,if i,j∉PP,  ei,j∈Ei,j=1,2,…,N.

Furthermore, matrix *A*_*N*×*N*_ is used to represent the reliability score matrix of* GW*, where the element *a*_*i*,*j*_ of *A*_*N*×*N*_ is defined as follows:

(2) $$\begin{array}{c} a_{i,j}=\left\{\begin{array}{cc} w\left(i,j\right), & \begin{array}{cc} & if\;e\left(i,j\right)\in E \end{array}\\ 0 & \begin{array}{cc} & if\;e\left(i,j\right)\notin E \end{array} \end{array}\right.\;i,j=1,2,\ldots ,N. \end{array}$$

If *a*_*i*,*j*_ ≥ *r*, we define the edge *e*(*i*, *j*) as a* r*-reliable link between nodes* i* and* j*, where* r* is a given reliability threshold and *r* ∈ {1,2,…, 999} [36].

Let *GE*_*N*×*T*_ denote the matrix of gene expression of* N* proteins across* T* time points. For a gene* i*, let *ev*_*i*,*t*_ represent the expression value of gene* i* at time* t* and *gep*_*i*_ = {*gep*_*i*_(*t*)∣*t* = 1,…, *T*} denote the gene expression pattern of gene* i*, where *gep*_*i*_(*t*) = (*ev*_*i*,*t*_ − *ev*_*min*_*i*_)/(*ev*_*max*_*i*_ − *ev*_*min*_*i*_), *ev*_*min*_*i*_⁡ = min_*t*=1_^*T*^⁡*ev*_*i*,*t*_, and *ev*_*max*_*i*_⁡=max_*t*=1_^*T*^⁡*ev*_*i*,*t*_,* i*=1,2,…,*N*,* t*=1,…,*T. *In fact, *gep*_*i*_ is composed of* T *normalized gene expression values. The normalized gene expression data can be used to measure the undulating degree above the base level of gene expression during a whole cell cycle.

### 2.2. Temporal PPI Networks

When a protein is involved in a specific biological process, the expression data of the protein-coding gene exhibits the undulation above the base level of the gene expression during the biological process. If the normalized expression value of gene* i *exceeds a specified threshold at a certain time point, we call that the product of gene* i* is activated at this time point. Let *ap*_*i*_(*t*) denote the active state of protein* i *at time point* t*, if protein* i* is active, *ap*_*i*_(*t*)=1, otherwise, *ap*_*i*_(*t*)=0,* i*=1,...,* N*, and* t*=1,...,* T*. For a given active threshold* φ*, *ap*_*i*_(*t*) is defined as follows:

(3) apit=1,if gepit≥φ0,if gepit<φ,i=1,…,N,  and  t=1,…,T.

Obviously, a lower active threshold* φ *preserves more gene dynamical information. The best active threshold* φ *will be discussed in the section “The effect of active threshold”.

In order to model the dynamics of active proteins mentioned above, the dynamical PPI network is introduced. In the following, we discuss how to construct temporal PPI networks (TPNs) by incorporating time-course gene expression data into static PPI network SPIN. Let *TPN*^*t*^ =(*V*^*t*^, *E*^*t*^) denote a temporal PPI network at time point* t*, where *V*^*t*^ and *E*^*t*^ is the set of proteins and the set of interactions between active proteins at time point* t*, respectively,* t*=1, 2,…,* T*. We use *DA*^*t*^ to represent the reliability score matrix of *TPN*^*t*^, where element *da*_*i*,*j*_^*t*^ in *DA*^*t*^ is computed as follows:

(4) dai,jt=apit×apjt×ai,j,i,j=1,…,N,  and  t=1,2,…,T

### 2.3. Temporal Interval PPI Networks

A protein complex is corresponding to a subgraph in PPI network. To represent the subgraph of a protein complex which appears in several successive temporal PPI networks, we introduce temporal interval PPI networks (TI-PINs). A temporal interval PPI network (TI-PIN) can be generated by merging several successive temporal PPI networks (TPNs). Given *TPN*^*t*^ =(*V*^*t*^, *E*^*t*^),* t*=1,...,*T*, let *TI*-*PIN*^*t*_*low*_,*t*_*top*_^ and *B*^*t*_*low*_,*t*_*top*_^ denote the temporal interval PPI network and its reliability score matrix from *t*_*low*_ to *t*_*top*_, respectively, where *t*_*low*_ and *t*_*top*_ are two time points and 1 ≤*t*_*low*_≤*t*_*top*_≤* T*. *TI*-*PIN*^*t*_*low*_,*t*_*top*_^ and element *b*_*i*,*j*_^*t*_*low*_,*t*_*top*_^ in *B*^*t*_*low*_,*t*_*top*_^ are defined as follows:

(5) TI-PINtlow,ttop=Vtlow,ttop,Etlow,ttop,Etlow,ttop=⋂t=tlowttopEt,1≤tlow≤ttop≤T

(6) bi,jtlow,ttop=if 1≤tlow=t=ttop≤Tmin⁡bi,jtlow,ttop−1,bi,jtlow+1,ttop,if 1≤tlow<ttop≤T,i,j=1,2,…,N

**Figure 1:**
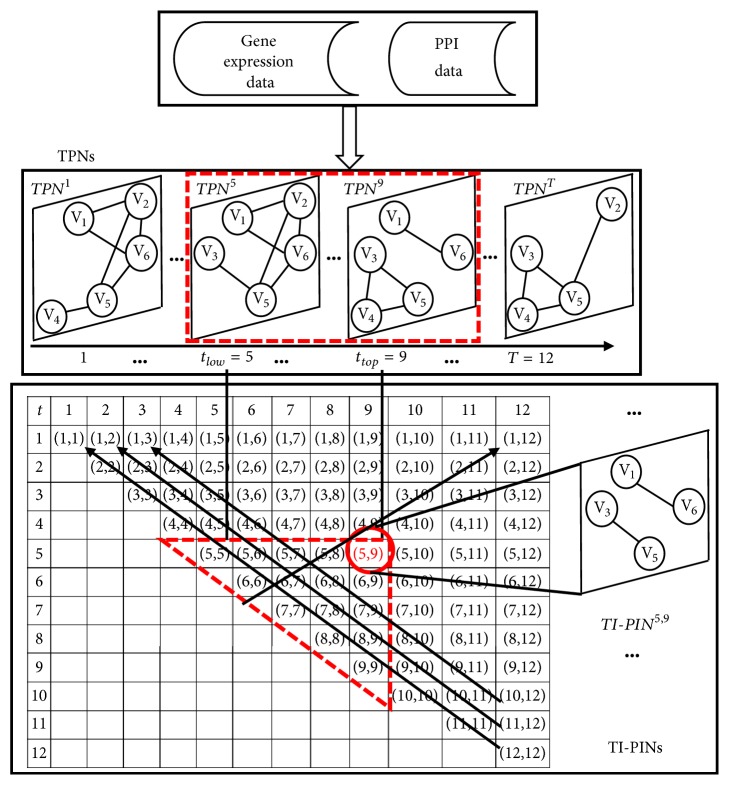
Generating *TI*-*PIN*^*t*_*low*_,*t*_*top*_^by merging the successive temporal PPI networks TPNs from *t*_*low*_ to *t*_*top*_. For example,* TI-PIN*^5,9^ is generated by merging TPNs from time point 5 to time point 9. TI-PINs in the triangle of red dash lines are generated by TPNs in the rectangle of red dash lines. A *TI*-*PIN*^*t*_1_,*t*_2_^ corresponds to a temporal interval (*t*_1_,* t*_2_), where 1 ≤* t*_1_ ≤* t*_2_ ≤* T *= 12.

Obviously, if 1 ≤*t*_*low*_* = t = t*_*top*_*≤ T*, then *b*_*i*,*j*_^*t*_*low*_,*t*_*top*_^=*b*_*i*,*j*_^*t*,*t*^=*da*_*i*,*j*_^*t*^, namely, *TI*-*PIN*^*t*,*t*^is the same as TPN^t^. If 1 ≤*t*_*low*_ < *t*_*top*_≤* T*, then*TI*-*PIN*^*t*_*low*_,*t*_*top*_^ is newly constructed by *TPN*^*t*^, *t*_*low*_ ≤ t ≤ *t*_*top*_. Here, let* l*=*t*_*top*_-*t*_*low*_+1 denote the temporal interval length.  Figure 1 shows the generation of *TI*-*PIN*^*t*_*low*_,*t*_*top*_^ by merging the successive temporal PPI networks (TPNs) from *t*_*low*_ to *t*_*top*_. From Figure 1, we can see that* T* TPNs can generate T·(*T*-1)/2 TI-PINs.

For given time points *t*_*low*_ and *t*_*top*_, and *t*_*low*_<*t*_*top*_, if the PPI subgraph *G*_*pc*_ of a protein complex appears in all *TPN*^*t*_*low*_^,*TPN*^*t*_*low*_+1^,…, and *TPN*^*t*_*top*_^, then *G*_*pc*_ also appears in all *TI*-*PIN*^*t*_1_,*t*_2_^,* t*_1_ ≤* t*_2_ and* t*_1_,* t*_2_*=t*_*low*_, *t*_*low*_+1,..., and *t*_*top*_. Because the number of *TI*-*PIN*^*t*_1_,*t*_2_^ is larger than the number of *TPN*^*t*^, the chance of exactly identifying the protein complex from *TI*-*PIN*^*t*_1_,*t*_2_^ is higher than that from *TPN*^*t*^, where* t*_1_ ≤* t*_2_ and* t*,* t*_1_,* t*_2_*=t*_*low*_, *t*_*low*_+1,..., *t*_*top*_.

### 2.4. Identification Method

In this section, we introduce the concepts of the joint colocalization condition, the joint coexpression condition, the GO-based functional similarity between proteins, and the expanding cluster condition and then present our identification method.

#### 2.4.1. Joint Colocalization Condition

To accomplish a specific biological process, some proteins physically interact with each other to form a protein complex at the same subcellular localization. Huh et al. [37] investigated the distribution of yeast proteins at different subcellular localization. Without loss of generality, we use yeast protein subcellular localization to illustrate the construction of joint colocalization condition of a protein complex. Yeast protein subcellular localization is classified into 22 categories shown in Table 1 [37]. Based on the subcellular localization category, a 22-dimension 0-1 vector is defined to represent the protein subcellular localization indicating the appearance of a protein during a whole cell cycle.

Given a protein* p*, let* LV*(*p*) denote the localization vector of the protein* p *and *LV*_*i*_(*p*) denote the* i*-th element of* LV*(*p*),* i*=1,…, 22. If the protein* p *is once localized at the* i*-th subcellular localization category in a whole cell cycle, *LV*_*i*_(*p*)=1, otherwise, *LV*_*i*_(*p*)=0,* i*=1,…, 22.

Given a set* PS *of* k* proteins {*p*_1_, *p*_2_,…, *p*_*k*_} and *LV*(*p*_*j*_),* j*=1,…,* k*, let* JLV*(*PS*)= {*JLV*_1_(*PS*), *JLV*_2_(*PS*),…, *JLV*_22_(*PS*)} denote the joint localization vector of* PS*, where *JLV*_*i*_(*PS*)= ∧_*j*=1_^*k*^*LV*_*i*_(*p*_*j*_),* i*=1,…,22, and “∧” is the logical AND operation on the elements among the localization vectors of proteins in* PS*. If all proteins in* PS* perform a specific function at the* i*-th subcellular localization category, then *JLV*_*i*_(*PS*)=1, otherwise, *JLV*_*i*_(*PS*)=0,* i*=1,…, 22. Obviously,* JLV*(*PS*) is also a 22-dimension 0-1 vector.

Given a set* PS* of proteins and its* JLV*(*PS*), let* JC*(*PS*)=∑_*i*=1_^22^*JLV*_*i*_(*PS*) denote the joint colocalization count of* PS*. Clearly,* JC*(*PS*) is the sum of all elements in* JLV*(*PS*). If* JC*(*PS*)>0, there exists at least one subcellular localization category where all proteins in* PS* are jointly colocalized in a whole cell cycle. If* JC*(*PS*)=0, all proteins in* PS* are not jointly colocalized at any subcellular localization category in a whole cell cycle. We define “*JC*(*PS*)>0” as the joint colocalization condition.

#### 2.4.2. Joint Coexpression Condition

There exists a correlation between gene expression level and protein activity [38]. The subunits in a permanent complex are coexpressed [39]. That suggests analyzing gene coexpression can reveal the potential interaction between active proteins to some extent.

Given a set* GS* of* k* genes {*g*_1_, *g*_2_,…, *g*_*k*_} and the normalized gene expression value* gep*_*i*_(*t*) of gene* i *at time point* t*,* t*=1,…,*T*,* i*=1,…,*k*, we use *JGE*_*GS*_={*JGE*_*GS*_(*t*)∣*t* = 1,…, *T*} to denote the joint gene expression profile of* GS*, where *JGE*_*GS*_(*t*) = ∏_*i*=1_^*k*^*gep*_*i*_(*t*) and “Π” is the multiplication operation on the expression pattern values among* k* genes. In essence, we can generate *JGE*_*GS*_(*t*) by calculating the product of the normalized expression values of* k* genes in* GS* at time point* t*,* t*=1,...,* T*.

To measure the joint coexpression level of* GS*, we use* JQ*(*GS*)=(1/*T*)∑_*t*=1_^*T*^*JGE*_*GS*_(*t*) to denote the joint coexpression quantity of* GS*. If* JQ*(*GS*)≥*γ*, all genes in* GS* are jointly coexpressed, where *γ* is the given threshold. We define “*JQ*(*GS*)≥*γ*” as the joint coexpression condition.

When the temporal interval length is* l*, we use* l*+4 successive expression data to analyze the joint coexpression condition. We set a time window, which covers* l*+4 successive time points, on the normalized expression data. If the current temporal interval is (*t*_*low*_, *t*_*top*_), the time window covers* l*+4 time points including *t*_*low*_-2, *t*_*low*_-1, *t*_*low*_,*...*, *t*_*top*_, *t*_*top*_+1, and *t*_*top*_+2. If *t*_*low*_ < 3, the time window consists of the following time points: 1, 2,..., *t*_*top*_, *t*_*top*_+1, and *t*_*top*_+2. If *t*_*top*_ > *T*-2, the time window consists of the following time points: *t*_*low*_-2, *t*_*low*_-1, *t*_*low*_,...,*T*-1, and* T*.

#### 2.4.3. The GO-Based Functional Similarity between Proteins

Ontology provides well-defined, structured, and computable semantics of domain knowledge [40]. Because of the need for consistent description related to genes and gene products across species, gene ontology GO has been launched by a collaborative effort to build complete ontologies in the biological domain [41]. GO terms include biological process (BP), molecular function (MF), and cellular component (CC). BP is referred to as a biological objective to which the gene or gene product contributes. MF is defined as the biochemical activity of a gene product. And CC is referred to as the place in the cell where a gene product is active [42]. These terms are semantically and hierarchically organized into a directed acyclic graph (DAG).

Semantic similarity is a function to measure closeness in meaning between ontological terms [43]. The GO semantic similarity score can be applied to quantify functional similarity between proteins. We compute the GO term based functional similarity *sim*_*go*_(*P*_1_,* P*_2_) between two proteins* P*_1_ and* P*_2_ by formula (7) [44, 45].

(7) simgoP1,P2=∑i=1mSimgo1,i,ST2+∑j=1nSimgo2,j,ST1m+n

where* ST*_1_={*go*_1,1_, *go*_1,2_,…, *go*_1,*m*_} is a term set annotating protein* P*_1_,* ST*_2_={*go*_2,1_, *go*_2,2_,…, *go*_2,*n*_} is a term set annotating protein* P*_2_, and* Sim*(*go*_1,*i*_,* ST*_2_) and* Sim*(*go*_2,*j*_,* ST*_1_) are computed by formula (8).

(8) $$\begin{array}{c} Sim\left(\;go,ST\;\right)=\overset{k}{\underset{j=1}{max}}\;sim\left(\;go,go_{j}\;\right) \end{array}$$

where* go* denotes a GO term,* ST*={*go*_1_, *go*_2_,…, *go*_*k*_} denotes a set of* k* GO terms, and* sim*(*go*, *go*_*j*_) is computed by formula (9).

(9) $$\begin{array}{c} sim\left(\;go_{1},go_{2}\;\right)=e^{-c_{1}l}\cdot \frac{e^{c_{2}h}-e^{-c_{2}h}}{e^{c_{2}h}+e^{-c_{2}h}}\cdot \frac{e^{c_{3}d}-e^{-c_{3}d}}{e^{c_{3}d}+e^{-c_{3}d}} \end{array}$$

where* go*_1_ and* go*_2_ are two different GO terms,* l *denotes the sum of the lengths of the shortest paths from* mica* to* go*_1_ and from* mica* to* go*_2_,* h *and* d* represent the depth and the information content of* mica*, respectively, and *c*_1_=0.2, *c*_2_=0.3, *c*_3_=30, while* mica* is referred to as the maximum informative common ancestor of two terms* go*_1_ and* go*_2_ in a DAG [44].

Correspondingly, we use formulas (7)-(9) to calculate the MF term based similarity *sim*_*mf*_(*P*_1_,* P*_2_), the CC term based similarity *sim*_*cc*_(*P*_1_,* P*_2_), and the BP term based similarity *sim*_*bp*_(*P*_1_,* P*_2_) between proteins* P*_1_ and* P*_2_, respectively. The values of *sim*_*mf*_(*P*_1_,* P*_2_), *sim*_*cc*_(*P*_1_,* P*_2_), and *sim*_*bp*_(*P*_1_,* P*_2_) range from 0.0 to 1.0. The larger these values are, the more similar proteins* P*_1_ and* P*_2_ are. If *sim*_*mf*_(*P*_1_,* P*_2_)≥*ω*, proteins* P*_1_ and* P*_2_ are judged to be similar based on the MF term, where* ω *is a given threshold. Similarly, if *sim*_*cc*_(*P*_1_,* P*_2_)≥*σ* and *sim*_*bp*_(*P*_1_,* P*_2_)≥*θ*, proteins* P*_1_ and* P*_2_ are judged to be similar based on the CC term and the BP term, respectively, where* σ* and* θ *are given thresholds.

#### 2.4.4. Expanding Cluster Condition

It is well known that members of a protein complex are similar to each other in functionality. In order to use the seed expanding strategy to mine a functional aggregation cluster from a PPI network, we devise an expanding cluster condition to judge whether a protein is functionally similar to a protein cluster (*PC*). Our method uses the expanding cluster condition to iteratively add the functionally similar proteins into the protein cluster* PC* to generate candidate protein complexes with functional homogeneity.

Given a protein cluster* PC* and a protein* u*, the CC term based minimal similarity* CC*(*PC*,*u*), the MF term based minimal similarity* MF*(*PC*,*u*), and the BP term based minimal similarity* BP*(*PC*,*u*) between* PC* and* u* are defined by formulas (10), (11), and (12), respectively.

(10) CCPC,u=min⁡simccu,v ∣ au,v≥r,v∈PC

(11) MFPC,u=min⁡simmfu,v ∣ au,v≥r,v∈PC

(12) BPPC,u=min⁡simbpu,v ∣ au,v≥r,v∈PC

where* r* is a given reliability threshold.

To judge whether* CC*(*PC*,*u*),* MF*(*PC*,*u*), and* BP*(*PC*,*u*) exceed their specified thresholds* σ*,* ω*, and* θ,* respectively, we define three Boolean variables* bcc*,* bmf*, and* bbp* as follows:

(13) bcc=true,if CCPC,u≥σfalse,otherwise

(14) bmf=true,if MFPC,u≥ωfalse,otherwise

(15) bbp=true,if BPPC,u≥θfalse,otherwise

If at least two out of three Boolean variables* bcc*,* bmf*, and* bbp* are “*true*” at the same time, the value of* EC*(*PC*,*u*) in formula (16) will become “*true*”. This means that the protein* u* is similar with the protein cluster* PC *in at least two aspects. Therefore, the protein* u *can be added into the protein cluster* PC*. We define “*EC*(*PC*,*u*)=*true*” as the expanding cluster condition.

(16) $$\begin{array}{c} EC\left(\;PC,u\;\right)=\left(\;bcc\wedge bmf\vee bcc\wedge bbp\vee bmf\wedge bbp\;\right) \end{array}$$

#### 2.4.5. Algorithm

The main idea of our algorithm is to iteratively construct temporal interval PPI network (TI-PIN) from time point* t*_1_ to time point* t*_2_, *TI*-*PIN*^*t*_1_,*t*_2_^ and identify protein complexes from *TI*-*PIN*^*t*_1_,*t*_2_^, 1 ≤* t*_1_ ≤* t*_2_ ≤* T*. To construct different temporal interval TI-PINs, our algorithm dynamically constructs TI-PINs in a bottom-up approach as shown in Figure 1. Firstly, the TI-PINs of temporal interval length* l*=1 are constructed. Next, the TI-PINs of temporal interval length* l*=2 are constructed, and so on. In Figure 1, the direction of arrow indicates the order of constructing TI-PINs. To identify a protein cluster, our algorithm initializes a protein cluster by selecting a node not being a member of any identified protein cluster, and successively checks the joint colocalization condition, the joint coexpression condition, and the expanding cluster condition to determine whether to add the adjacent nodes into the protein cluster, and terminates until no nodes around the protein cluster satisfy all three above-mentioned conditions. By repeating the identifying process of a protein cluster, different protein clusters (*PCs*) are identified one by one from the constructed TI-PINs. We call our algorithm as ICJointLE-DPN (Identifying protein** c**omplexes with the features of joint colocalization and joint coexpression from Dynamic Protein Networks).  Algorithm 1 describes ICJointLE-DPN in detail.

By converting temporal PPI networks TPNs into temporal interval PPI networks TI-PINs, the constructed TI-PINs preserve only interactions lasting over the temporal interval. Besides, the amount of the constructed TI-PINs is more than that of TPNs. So, our constructed TI-PINs can offer more opportunities to accurately identify more protein complexes.

Now we analyze the time complexity of ICJointLE-DPN. Consider Algorithm 1, ICJointLE-DPN dynamically constructs TI-PINs. For* T* time points, ICJointLE-DPN can construct* T∙*(*T*+1)/2 TI-PINs. For each constructed TI-PIN, there are at most* N* protein nodes, where* N* is the total number of protein nodes in the constructed TI-PIN. For each protein node not being a member of any identified protein cluster, ICJointLE-DPN selects this protein node as an initial protein cluster and expands the protein cluster by checking* N*-1 other protein nodes. The time complexity of identifying protein complexes from each constructed TI-PIN is* O*(*N∙*(*N*-1)), namely,* O*(*N*^2^). Therefore, the time complexity of ICJointLE-DPN is* O*(*N*^2^*∙T∙*(*T*+1)/2)=*O*((*N∙T*)^2^).

In the following section, we evaluate our constructed TI-PINs and other dynamic PPI networks and compare our proposed identification method with other competing methods.

## 3. Experiments and Results

In this section, we first introduce the testing data sets and the benchmark data. Subsequently, we describe metrics evaluating the quality of identified protein complexes. Finally, we present the experimental results and comparative analysis.

### 3.1. Experimental Dataset

To construct temporal interval PPI networks (TI-PINs), we selected three yeast PPI data sets. The first one, downloaded from the STRING database V10 version [36], consists of 6418 proteins and 939998 interactions with reliability score. The second one containing 5811 proteins and 256516 interactions was downloaded from the BioGrid database 3.4.128 version [46]. The last one, containing 5022 proteins and 22381 interactions, was downloaded from the DIP database with the release date 2015/07/01[47]. According to formula (1), we used reliability scores annotating interactions in STRING to score the interactions shared in STRING and BioGrid/DIP.

Furthermore, we selected two yeast gene expression data sets to conduct the comparative experiment. One data set, GSE3431 [48], is extracted from the file GDS2267_full.soft which was acquired with access number GDS2267 on http://www.ncbi.nlm.nih.gov/sites/GDSbrowser. GSE3431 is an expression profile of yeast by Affymetrix Yeast Genome S98 Array over three successive metabolic cycles. GSE3431 contains 36 raw gene expression data gathered at 25-minute interval. Let* T*_1_,* T*_2_,..., and* T*_36_ denote the 36 successive time points, thus we can calculate the average value* ave*_*ge*_*i*_ of three raw gene expression data at three time points *T*_*i*_, *T*_*i*+12_, and *T*_*i*+24_ for each gene in GSE3431. The average value* ave*_*ge*_*i*_ is used to represent the* i*-th gene expression value,* i*=1, 2,..., 12. We used the 12 gene expression values for each gene to analyze joint coexpression condition and construct TI-PINs for GSE3431. Another data set GSE4987 [49] is composed of gene expression data of wild type W303a cells, which are sampled at 5-minute interval over two hours per cell cycle across two cell cycles. GSE4987 contains 50 raw gene expression data across two cell cycles, where there are 25 raw gene expression data per cell cycle. Similarly, we calculated 25 gene expression values for each gene in GSE4987, and used the 25 gene expression values for each gene to analyze joint coexpression condition and construct TI-PINs for GSE4987.

In addition, we used the yeast-related protein localization data [37], downloaded from http://yeastgfp.yeastgenome.org, to analyze joint colocalization condition. The GO term annotations of the yeast-related proteins were downloaded from http://www.ncbi.nlm.nih.gov/geo/query/acc.cgi?acc=GSE3431. We used the GO term annotations to calculate the GO term based functional similarity between proteins. The known complexes set CYC2008 containing 408 manually curated heterometric protein complexes was downloaded from http://wodaklab.org/cyc2008/ [50].

### 3.2. Evaluation Metrics

Comparing identified complexes with known ones is a commonly used evaluation. There are several statistical matching-based metrics, which evaluate the quality of identified complexes and the performance of identification methods. The biological relevance-based metrics, which are independent of the known complexes, are used to evaluate the biological significance of identified complexes.

#### 3.2.1. The Statistical Matching-Based Metrics

If an identified complex and a known complex overlap each other, there exist common proteins between them. The overlapping score between an identified complex* ic *and a known complex* kc*,* OS*(*ic*,* kc*), is calculated as follows [4]:

(17) $$\begin{array}{c} OS\left(\;ic,kc\;\right)=\frac{{\left|V_{ic}\cap V_{kc}\right|}^{2}}{\left|V_{ic}\right|\times \left|V_{kc}\right|} \end{array}$$

where *V*_*ic*_ and *V*_*kc*_ are the protein set of* ic* and the protein set of* kc* respectively. If* OS*(*ic*,* kc*)≥*λ*,* ic *is matched with* kc*, where the threshold* λ* is usually set to 0.2 [4, 11]. Particularly,* OS*(*ic*,* kc*)=1 indicates that* ic* is completely matched with* kc*.

Let* IC* be the set of complexes identified by computational method and* KC* be the set of the known complexes. Then let* Mic *represent the number of identified complexes which matches at least one known complex in* KC*, and* Mkc *denote the number of known complexes which matches at least one identified complex in* IC*.* Mic* and* Mkc* are defined as follows [4]:

(18) Mic=ic ∣ ic∈IC,  ∃kc∈KC,  OSic,kc≥λ

(19) Mkc=kc ∣ kc∈KC,  ∃ic∈IC,  OSic,kc≥λ

We evaluate the quality of identified complexes by precision (*prec*), recall(*rec*), and f-measure (*fm*) [51].

(20) prec=MicIC

(21) rec=MkcKC

(22) fm=2×prec×recprec+rec

*Frac* is defined as the fraction of matched complexes, which measures the percentage of known complexes matched with identified complexes [5]. In fact,* Frac* is equivalent to* rec*.

The maximum matching ratio (*MMR*) [5] is based on a maximal one-to-one mapping between identified complex and known complex, and it measures accuracy that the identified complexes can represent the known complexes.* MMR* is calculated as follows [5]:

(23) $$\begin{array}{c} MMR=\frac{\sum_{i=1}^{n}\max\left\{OS\left(kc_{i},ic_{j}\right)\;|\;j=1,\ldots ,m\right\}}{n} \end{array}$$

where *kc*_*i*_ is the* i*-th known complex,* i*=1,...,*n*, and* n*= |*KC*|, and *ic*_*j*_ is the* j*-th identified complex,* j*=1,...,*m* and* m*= |*IC*|.

Let *n*_*i*_ be the number of proteins in the* i*-th known complex and *t*_*ij*_ be the number of common proteins between the* i*-th known complex and the* j*-th identified complex,* i*=1,…,*n*, and* j*=1,…,*m*. Sensitivity (*Sn*), positive predictive value (*PPV*), and geometric mean (*Acc*) of* Sn* and* PPV *[51] are used to assess the accuracy of identification methods.* Sn*,* PPV*, and* Acc* are computed by formulas (24)-(26).

(24) Sn=∑i=1nmax⁡tij ∣ j=1,2,…,m∑i=1nni

(25) PPV=∑j=1mmax⁡tij ∣ i=1,2,…,n∑i=1n∑j=1mtij

(26) Acc=Sn×PPV

As a result, the performance of identification method is evaluated by the comprehensive score* FAM*, which is calculated by formula (27) [5].

(27) $$\begin{array}{c} FAM=Frac+Acc+MMR \end{array}$$

Obviously,* FAM* is a metric measuring statistical match and is mainly used to statistically evaluate the identification accuracy.

Let #*PM *be the number of identified complexes that match with known complexes exactly. In fact, #*PM* is a metric for evaluating the degree of exact match between the identified complexes and known complexes.

In the following, we will illustrate how to use both #*PM* and* FAM* to comprehensively compare the quality of two sets of identified complexes via analyzing the relative performance of these two sets of identified complexes.

For two sets of identified complexes with metrics #*PM* and* FAM*,* S*_1_ with #*PM*_1_ and* FAM*_1_ and* S*_2_ with #*PM*_2_ and* FAM*_2_, let* G*_1,2_ denote the geometric mean of the relative performances of* S*_1_ and* S*_2_, and* G*_1,2_ is calculated as follows:

(28) $$\begin{array}{c} G_{1,2}=\sqrt{\frac{\#PM_{1}}{\#PM_{2}}\times \frac{FAM_{1}}{FAM_{2}}}=\sqrt{\frac{\#PM_{1}\times FAM_{1}}{\#PM_{2}\times FAM_{2}}} \end{array}$$

If* G*_1,2_> 1, then the quality of* S*_1_ will be superior to that of* S*_2_ in terms of the product of #*PM* and* FAM*; otherwise, the quality of* S*_2_ will be superior to that of* S*_1_ in terms of the product of #*PM* and* FAM*. Hence, whether the quality of a set of identified complexes is superior to that of another set of identified complexes can be judged in terms of the product of #*PM* and* FAM*. In essence, we treat the product of #*PM* and* FAM *as a comprehensive score of exact match and statistical match. So, in our experiments, we chose the product of #*PM* and* FAM*, #*PM*×*FAM*, as the major metric to comprehensively evaluate the quality of identified complexes.

#### 3.2.2. The Biological Relevance-Based Metrics

We noticed that the known complexes are generally incomplete [52]. Even though an identified complex does not match with any known complex, it may be an uncharacterized but valid complex [5]. A protein complex tends to be responsible for a specific biological process or molecular function [53]. Hence, it is necessary for evaluating biological relevance to analyze the over-expression of an identified protein complex.

The GO term based over-expression analysis for biological process and molecular function can be used to reveal functional homogeneity of proteins in a complex to some extent [5]. For a PPI network containing* N* proteins, we use* K* to denote the total number of the term* X*-annotated proteins in the PPI network. For a given complex containing *n*_*s*_ proteins, the* p-value *of this complex is defined as the probability that the number of term* X*-annotated proteins in a protein set of size *n*_*s*_ is not less than *k*_*s*_, where *k*_*s*_ is the number of the term* X*-annotated proteins in this complex [54]. The* p-value* is computed as follows [54]:

(29) $$\begin{array}{c} p\text{-}value=1-\overset{k_{s}-1}{\underset{i=0}{\sum }}\frac{\left(\begin{array}{c} N-K\\ n_{s}-i \end{array}\right)\left(\begin{array}{c} K\\ i \end{array}\right)}{\left(\begin{array}{c} N\\ n_{s} \end{array}\right)}=\overset{n_{s}}{\underset{i=k_{s}}{\sum }}\frac{\left(\begin{array}{c} N-K\\ n_{s}-i \end{array}\right)\left(\begin{array}{c} K\\ i \end{array}\right)}{\left(\begin{array}{c} N\\ n_{s} \end{array}\right)} \end{array}$$

We used the open source software GO::TermFinder [55] to calculate the* p*-*value* of an identified complex.

If* p-value*<*ψ*, we call that the term* X*-annotated proteins enrich the complex at* ψ*-level [54], where* ψ *is a given threshold. If the term* X*-annotated proteins enrich a complex at the level of* ψ*=0.01 [54], this complex will has significantly biological function and be called significant complex [5]. The over-expression score of a set of identified protein complexes is defined as the ratio of the number of significant protein complexes to the total number of protein complexes in the set. We can evaluate the biological relevance of a set of identified protein complexes by calculating its over-expression score.

### 3.3. Experimental Results

Firstly, we evaluated the effect of active threshold* φ* on the quality of protein complexes identified from temporal PPI networks (TPNs). Secondly, we assessed the protein complexes identified from temporal interval PPI networks (TI-PINs). Finally, we compared our method ICJointLE-DPN with Zhang's method [33], DPC-NADPIN [34], TS-OCD [31], and DCA [35].

#### 3.3.1. The Effect of Active Threshold

**Figure 2:**
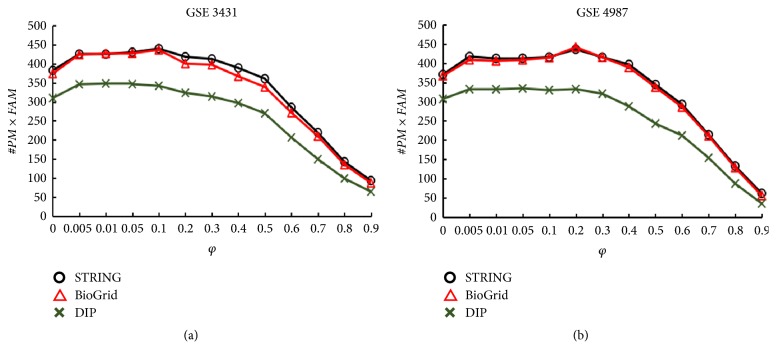
Plot of value of #*PM*×*FAM *of complexes identified from the constructed TPNs by integrating gene expression data into yeast PPI data with respect to value of* φ.*(a) Plot of value of #*PM*×*FAM *of complexes identified from the constructed TPNs by integrating GSE3431 into STRING, BioGrid, and DIP, respectively, with respect to value of* φ*. (b) Plot of value of #*PM*×*FAM *of complexes identified from the constructed TPNs by integrating GSE4987 into STRING, BioGrid, and DIP, respectively, with respect to value of* φ*.

Here we first constructed different temporal PPI networks (TPNs) by combining three yeast PPI data sets (STRING, BioGrid, and DIP) with two yeast gene expression data sets (GSE3431 and GSE4987). And then we evaluated the quality of the complexes identified from these different TPNs.  Figure 2 shows the variation curves of value #*PM*×*FAM* of complexes identified from different constructed TPNs with the changing* φ*.

From Figure 2(a) we can see that for GSE3431, the value of #*PM*×*FAM* of the complexes identified from the constructed TPNs is the largest when* φ*=0.01 for DIP and* φ*=0.1 for STRING and BioGrid respectively. At the meantime, from Figure 2(b), we can also see that for GSE4987, the value of #*PM*×*FAM *is the largest when* φ*=0.05 for DIP, and the value of #*PM*×*FAM *is the largest when* φ*=0.2 for STRING and BioGrid. Hence, in the following experiments, these values of* φ*, shown in Table 2, are used to construct different TPNs for different combination of yeast expression data sets and yeast PPI data sets.

#### 3.3.2. Setting of Parameters for ICJointLE-DPN

In our experiments, we empirically adjusted the value of parameters* σ*,* ω*, and* θ* to enable ICJointLE-DPN to perform well. We adjusted the value of parameters* σ*,* ω*, and* θ *from 0.1 to 0.9 by increment 0.1 through several experiments respectively, and set these parameters to the appropriate values.

Table 3 shows the values of four parameters for algorithm ICJointLE-DPN with different combination of yeast gene expression data sets and yeast PPI data sets.

#### 3.3.3. Evaluating Identified Complexes

To evaluate the quality of complexes identified by our method ICJointLE-DPN, we first constructed TPNs and TI-PINs. And then we executed algorithm ICJointLE-DPN to identify complexes from SPIN, TPNs, and TI-PINs respectively. Finally, we compared the quality of the complexes identified from SPIN, TPNs, and TI-PINs respectively in terms of value of #*PM*×*FAM*, which is shown in Table 4.

As seen in Table 4, we can find that for the same yeast PPI data set, the values of #*PM*×*FAM* resulting from both TPNs and TI-PINs are apparently larger than that resulting from SPIN. This indicates that identifying protein complexes from dynamic PPI networks can improve the quality of identified complexes. From Table 4, we can also see that the value of #*PM*×*FAM *resulting from TI-PINs is larger than that from TPNs. It means that identifying protein complexes from TI-PINs can further enhance the quality of identified complexes. As mentioned in the section “temporal interval PPI networks”, the use of TI-PINs constructed by several successive TPNs can provide more opportunities to accurately identify more protein complexes.

**Figure 3:**
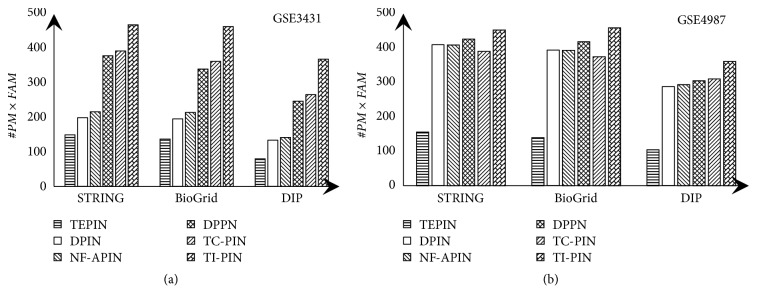
Comparison of values of #*PM*×*FAM *of complexes identified by ICJointLE-DPN from different dynamic PPI networks. (a) GSE3431 and (b) GSE4987 are integrated into STRING, BioGrid, and DIP, respectively, to construct six types of dynamic protein interaction networks.

To further illustrate the effect of our constructed TI-PINs, we ran our algorithm ICJointLE-DPN to identify complexes from TI-PINs and other existing dynamic PPI networks respectively. The experimental results are shown in Figure 3.

We can see from Figure 3 that no matter which yeast PPI data set is integrated with either GSE3431 or GSE4987 to construct TI-PINs, the value of #*PM*×*FAM *of complexes identified by ICJointLE-DPN from the constructed TI-PINs is apparently larger than that from other dynamic PPI networks. Such results may partly be attribute to using the relatively low active threshold* φ.* In addition, by preserving continuous interactions, our constructed TI-PINs can indeed offer more opportunities to identify more protein complexes accurately.

As a result, our constructed TI-PINs have more contributions to identification of protein complexes than other dynamic PPI networks such as TEPIN, DPIN, NF-APIN, DPPN, and TC-PINs.

#### 3.3.4. Comparing Identification Methods

In order to evaluate the performance of the identification methods, we compared our method ICJointLE-DPN to three other competing methods Zhang's method [33], DPC-NADPIN [34], TS-OCD [31], and DCA[35]. As described in the section “Expanding cluster condition”, in our method, only those PPIs with reliability score not lower than reliability threshold* r* are used to identify protein complexes. For fair comparison, we removed those PPIs with reliability score lower than reliability threshold* r* in three yeast PPI data sets before executing four other competing methods. For DPC-NADPIN method, no parameters need to be set. Zhang's method uses two parameters* Pre*_*thresh *and* Complex*_*thresh *whose default values are 0.5 and 0.1. For DCA, we set parameters to the recommended values *α*=0.6, *β*=0.55, and *γ*=1.4. The setting of nine parameters used in TS-OCD method is shown in Table 5.

By analyzing known complexes in CYC2008, we found that the number of the complexes of size two to six exceeds 84% of the total number of known complexes. To evaluate the ability of identifying complexes of size two to six, Table 6 shows the distribution of the size of the complexes identified exactly by five methods.

From Table 6, we can see that our method ICJointLE-DPN has stronger ability of exactly identifying the complexes of size two to six than other four competing methods. Especially, DPC-NADPIN, TS-OCD, and DCA fail to identify any complexes of size two.

To evaluate the overall performance of five competing methods, we reported the statistical matching-based metrics of the identified complexes in Table 7.

From Table 7, we can see that our method ICJointLE-DPN outperforms the other four competing methods in terms of* #PM*,* Frac*,* MMR*,* FAM*, and* #PM×FAM*. We also see that, concerning* fm*, ICJointLE-DPN obtains almost all the largest values except for one among five competing methods, and with regard to* Acc*, ICJointLE-DPN is ranked top two. Overall, our method ICJointLE-DPN can not only identify complexes accurately but also identify more complexes exactly matched with known complexes from TI-PINs.

**Figure 4:**
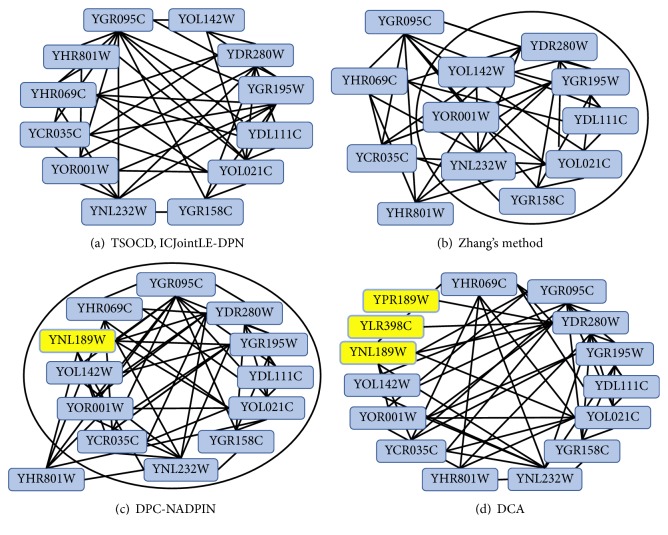
The complexes identified by five competing methods. (a) The No. 396 complex identified by TSOCD and the No. 901 complex identified by ICJointLE-DPN both match with nuclear exosome complex exactly. (b) The No. 829 complex is incorrectly identified by Zhang's method by not including the four proteins outside the ellipse. (c) The No. 1929 complex identified by DPC-NADPIN by wrongly including the yellow-colored YNL189W and excluding YHR081W. (d) DCA identified the No. 55 complex wrongly with the inclusion of three yellow-colored proteins.

Now we give two examples related to the complexes identified from dynamical PPI networks which are constructed via incorporating GSE3431 into DIP.  Figure 4 illustrates the matching example between nuclear exosome complex and the complexes identified by five competing methods.

As can be seen from Figure 4(a), TSOCD and ICJointLE-DPN can identify nuclear exosome complex exactly. Zhang's method misses four proteins outside the ellipse in Figure 4(b). DPC-NADPIN wrongly identifies the yellow-colored YNL189W and misses YHR081W outside the ellipse in Figure 4(c). DCA wrongly identifies three yellow-colored proteins in Figure 4(d).

**Figure 5:**
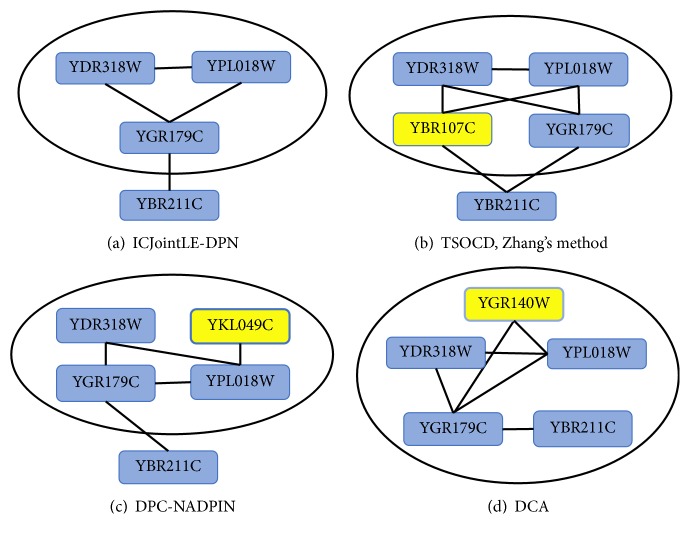
The complexes identified by five competing methods. (a) The No. 1935 complex identified by ICJointLE-DPN misses YBR211C outside the ellipse. (b) The No. 123 complex identified by TSOCD and the No. 137 complex identified by Zhang's method incorrectly contain the yellow-colored YBR107C and omit YBR211C outside the ellipse. (c) The No. 159 complex identified by DPC-NADPIN wrongly includes the yellow-colored YKL049C and omits YBR211C outside the ellipse. (d) The No. 433 complex identified by DCA wrongly includes the yellow-colored YGR140W.

Similarly, Figure 5 shows the matching example between COMA complex and the complexes identified by five competing methods.

From Figure 5, we can see that our method ICJointLE-DPN fails to identify COMA complex exactly due to missing YBR211C outside the ellipse in Figure 5(a). TSOCD and Zhang's method wrongly identify the yellow-colored YBR107C and miss YBR211C outside the ellipse in Figure 5(b), these two methods are unable to detect COMA complex exactly. Likewise, owing to wrongly identifying the yellow-colored YKL049C and missing YBR211C outside the ellipse in Figure 5(c), DPC-NADPIN fails to find COMA complex exactly. We can also see from Figure 5(d) that DCA is unsuccessful in detecting the COMA complex due to wrongly identifying the yellow-colored YGR140W.

To evaluate the functional enrichment of identified complexes, we compared our method ICJointLE-DPN to other four competing methods with respect to biological process (BP) enrichment analysis. For complexes identified by ICJointLE-DPN from different TI-PINs, their raw data of BP enrichment analyses and their results of significant statistics are presented in Supplementary Materials (Available here).  Table 8 shows the proportion of the complexes that are significantly enriched by BP term-annotated proteins, where #*IC *is the total number of identified complexes, #*SC *denotes the number of identified complexes with significant enrichment.

As seen from Table 8, for five competing methods, their identified complexes of size larger than 6 are almost biologically significant except for the four italic cases. From Table 8, we can also see that for the significant enrichment of identified complexes of size not larger than 6, our method performs slightly weaker than DPC-NADPIN, TS-OCD, and DCA but stronger than Zhang's method.

In summary, our proposed identification method overall outperforms other four competing methods in terms of the number of identified complexes exactly matched with known complexes* #PM*, the fraction of known complexes matched with identified complexes* FRAC*, maximum matching ratio* MMR*, comprehensive score* FAM*, and the product of* #PM *and* FAM.* Concerning the significant enrichment, five competing methods overall perform well when they identify complexes of size larger than 6; when identifying complexes of size not larger than 6, our proposed method performs slightly weaker than DPC-NADPIN, TS-OCD, and DCA but stronger than Zhang's method.

## 4. Conclusions

Gene expression data contains temporal information of protein activity. By integrating gene expression data into PPI data to determine active time point of interacting proteins, we exploited temporal dynamics of proteins to construct temporal PPI networks TPNs. In order to accurately identify more protein complexes, we further converted TPNs into temporal interval PPI networks TI-PINs. The experimental results confirmed that our constructed TI-PINs have more contributions to identification of protein complex than TEPIN (Time-Evolving PIN), DPIN (dynamic protein interaction networks), NF-APIN (noise-filtered active protein interaction networks), DPPN (dynamic probabilistic protein interaction networks), and TC-PIN (time-course protein interaction networks).

Based on our constructed TI-PINs, we devised a novel method ICJointLE-DPN which uses multisource biological data to identify protein complexes. First, our proposed method employs protein localization data to analyze the joint colocalization condition to judge whether a group of proteins is of joint colocalization. Secondly, our proposed method uses gene expression data to analyze the joint coexpression condition to judge whether a group of proteins is of joint coexpression. Thirdly, our method exploits three types of similarity to analyze the expanding cluster condition to judge whether a group of proteins is of functional homogeneity. As a result, by combining these three conditions, our proposed method can accurately identify more protein complexes from TI-PINs than other four competing methods Zhang's method, DPC-NADPIN, TS-OCD, and DCA.

Identifying protein complexes from dynamic PPI networks remains to be a challenging work in postgenomic era. In cell system, protein activity and protein-protein interaction have dynamical characteristics. Hence, it is important for identifying protein complexes to construct dynamic PPI networks close to reality. Due to the limited gene expression samples and failure to capture some transient interactions, it is difficult to construct dynamic PPI networks completely expressing protein interactions in cell system. Although many works have made to construct effective dynamic PPI networks to identify protein complexes, the efforts on constructing nearly real PPI networks will still be encouraged. In addition, it is also important to design an effective method to identify protein complexes from dynamic PPI networks. To find protein complexes with biological relevance by computational approach, multisource biological data should be used to identify protein complexes from dynamic PPI networks. As seen from Table 8, some protein complexes of size not larger than 6 identified by our method are not significant enough in biological meaning. This suggests that more other biological data should be integrated into protein complex identification. In future work, we will further investigate the integration of more biological data into our method in order to not only identify protein complexes more accurately but also improve the significant enrichment of the identified protein complexes of size not larger than 6.

## Figures and Tables

**Algorithm 1:**
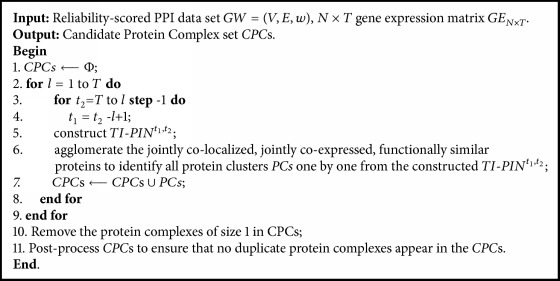
ICJointLE-DPN.

**Table 1:** Subcellular localization category for yeast subcellular compartment.

No.	subcellular localization category	No.	subcellular localization category	No.	subcellular localization category	No.	subcellular localization category
1	mitochondrion	7	ER	13	late Golgi	19	early Golgi
2	vacuole	8	nuclear periphery	14	peroxisome	20	lipid particle
3	spindle pole	9	endosome	15	actin	21	nucleus
4	cell periphery	10	bud neck	16	nucleolus	22	bud
5	punctate composite	11	microtubule	17	cytoplasm		
6	vacuolar membrane	12	Golgi	18	ER to Golgi		

*Note*. No. is the subcellular localization category number.

**Table 2:** Setting values of *φ* for different combination of yeast gene expression data sets and yeast PPI data sets.

Yeast expression data sets	Yeast PPI data sets	*φ*
GSE3431	STRING	0.1
	BioGrid	0.1
	DIP	0.01

GSE4987	STRING	0.2
	BioGrid	0.2
	DIP	0.05

**Table 3:** Value of parameters* r*, *σ*, *ω*, and *θ* for ICJointLE-DPN.

Yeast expression data sets	Yeast PPI data sets	r	*σ*	*ω*	*θ*
GSE3431	STRING	999	0.7	0.6	0.1
	BioGrid	999	0.7	0.6	0.1
	DIP	990	0.7	0.1	0.3

GSE4987	STRING	999	0.7	0.6	0.1
	BioGrid	999	0.7	0.6	0.1
	DIP	990	0.7	0.1	0.3

**Table 4:** Values of #*PM*×*FAM* of complexes identified from SPIN, TPNs, and TI-PINs.

Yeast expression data sets	Yeast PPI networks	#PM×FAM		
		STRING	BioGrid	DIP
GSE3431	SPIN	356.54	337.93	282.01
	TPNs	433.68	415.28	347.72
	TI-PINs	*441.38*	*423.30*	*356.69*

GSE4987	SPIN	338.49	334.74	277.48
	TPNs	424.25	416.00	343.00
	TI-PINs	*426.60*	*420.18*	*345.02*

**Table 5:** Setting parameters for TS-OCD method.

Parameters	*Repeat_times*	*tau*	*delta*	*T*	*K*	*lambda*	*beta*	*iter*	*rho*
value	1	0.3	0.3	12	1000	0.0625	16	20	0.000001

**Table 6:** Distribution of the size of the exactly identified complexes.

Yeast expression data sets	Yeast PPI Data sets	Methods	The number of the exactly identified complexes													Total
			Size	2	3	4	5	6	7	8	9	10	12	14	15	
GSE3431	STRING	ICJointLE-DPN		*112*	*44*	*17*	*7*	*4*	1	3	1	0	1	0	0	*190*
		Zhang's method		53	19	9	5	2	1	2	0	0	1	0	0	92
		DPC-NADPIN		0	18	10	4	0	1	0	0	0	1	0	0	34
		TS-OCD		0	5	7	3	1	1	1	0	0	1	1	0	20
		DCA		0	4	3	0	0	0	0	0	0	0	0	0	7
	BioGrid	ICJointLE-DPN		*113*	*42*	*15*	*6*	*4*	0	1	2	1	1	0	0	*185*
		Zhang's method		56	20	10	5	3	1	2	0	0	1	0	0	98
		DPC-NADPIN		0	20	11	4	1	1	1	0	0	1	0	0	39
		TS-OCD		0	19	7	4	4	2	3	0	0	1	0	1	41
		DCA		0	4	3	1	0	0	0	0	0	0	0	0	8
	DIP	ICJointLE-DPN		*111*	*41*	*11*	*4*	*2*	1	1	2	0	1	0	0	*174*
		Zhang's method		65	17	7	2	1	1	1	0	0	0	0	0	94
		DPC-NADPIN		0	11	6	2	1	1	1	1	0	0	0	0	23
		TS-OCD		0	4	5	0	1	1	0	0	0	1	0	0	12
		DCA		0	3	5	1	0	0	0	0	0	0	0	0	9

GSE4987	STRING	ICJointLE-DPN		*107*	*45*	*17*	*6*	*5*	0	2	1	1	0	1	0	*185*
		Zhang's method		52	21	12	4	3	2	2	0	0	0	0	1	97
		DPC-NADPIN		0	3	1	2	0	0	0	0	0	0	0	0	6
		TS-OCD		0	2	2	4	3	1	2	0	1	0	0	0	15
		DCA		0	2	2	1	1	0	0	0	0	0	0	0	6
	BioGrid	ICJointLE-DPN		*111*	*46*	*16*	*4*	*4*	0	1	3	2	0	0	0	*187*
		Zhang's method		59	22	13	4	3	2	1	0	0	0	0	0	104
		DPC-NADPIN		0	4	2	2	0	0	0	0	0	0	0	0	8
		TS-OCD		0	16	7	5	5	2	2	0	1	0	1	0	39
		DCA		0	2	3	2	1	0	1	0	0	0	0	0	9
	DIP	ICJointLE-DPN		*110*	*40*	*13*	*4*	*2*	1	1	2	1	0	0	0	*174*
		Zhang's method		69	20	8	3	3	2	1	0	0	0	0	0	106
		DPC-NADPIN		0	4	2	0	0	0	0	0	0	0	0	0	6
		TS-OCD		0	8	3	2	2	1	0	0	0	0	0	0	16
		DCA		0	5	3	2	1	0	0	0	0	0	0	0	11

**Table 7:** Statistical matching-based metrics of the complexes identified by five competing methods.

Yeast expression data sets	Yeast PPI data sets	Methods	#*PM*	*fm*	*Frac*	*Acc*	*MMR*	*FAM*	*#PM×FAM*
GSE3431	STRING	ICJointLE-DPN	*190*	*0.71*	*0.95*	0.75	*0.75*	*2.45*	*465.50*
		Zhang's method	92	0.63	0.86	*0.80*	0.59	2.25	207.00
		DPC-NADPIN	34	0.71	0.77	0.71	0.48	1.96	66.64
		TS-OCD	20	0.55	0.46	0.68	0.30	1.45	29.00
		DCA	7	0.55	0.51	0.57	0.27	1.35	9.45
	BioGrid	ICJointLE-DPN	*185*	*0.72*	*0.93*	*0.80*	*0.73*	*2.46*	*455.10*
		Zhang's method	98	0.63	0.85	0.78	0.58	2.21	216.58
		DPC-NADPIN	39	0.71	0.75	0.72	0.48	1.94	75.66
		TS-OCD	41	0.66	0.65	0.73	0.43	1.81	74.21
		DCA	8	0.55	0.51	0.56	0.27	1.34	10.72
	DIP	ICJointLE-DPN	*174*	*0.69*	*0.85*	0.70	*0.68*	*2.23*	*388.02*
		Zhang's method	94	0.67	0.76	*0.71*	0.52	1.99	187.06
		DPC-NADPIN	23	0.63	0.55	0.67	0.35	1.57	36.11
		TS-OCD	12	0.39	0.27	0.54	0.18	1.00	12.00
		DCA	9	0.48	0.36	0.55	0.21	1.12	10.08

GSE4987	STRING	ICJointLE-DPN	*185*	0.60	*0.95*	0.72	*0.75*	*2.42*	*447.70*
		Zhang's method	97	*0.64*	0.91	*0.79*	0.62	2.32	225.04
		DPC-NADPIN	6	0.52	0.56	0.59	0.30	1.46	8.76
		TS-OCD	15	0.55	0.53	0.68	0.34	1.56	23.4
		DCA	6	0.61	0.70	0.64	0.37	1.71	10.26
	BioGrid	ICJointLE-DPN	*187*	*0.67*	*0.93*	0.76	*0.74*	*2.43*	*454.41*
		Zhang's method	104	0.63	0.90	*0.78*	0.63	2.31	240.24
		DPC-NADPIN	8	0.54	0.55	0.60	0.13	1.45	11.6
		TS-OCD	39	0.66	0.68	0.74	0.46	1.88	73.32
		DCA	9	0.62	0.68	0.65	0.36	1.69	15.17
	DIP	ICJointLE-DPN	*174*	*0.67*	*0.86*	0.68	*0.68*	*2.22*	*386.28*
		Zhang's method	106	0.67	0.83	*0.74*	0.58	2.15	227.9
		DPC-NADPIN	6	0.44	0.36	0.53	0.21	1.10	6.60
		TS-OCD	16	0.41	0.29	0.54	0.20	1.04	16.64
		DCA	11	0.58	0.50	0.63	0.29	1.42	15.62

**Table 8:** Proportion of the complexes enriched significantly by BP term-annotated proteins.

Yeast expression data sets	Yeast PPI data sets	Methods	*#IC*	*#SC*	*% of significant (size≤6)*	*% of significant (6<size<20)*	*% of significant (size≥20)*
GSE3431	STRING	ICJointLE-DPN	5137	4873	93.33%	100.00%	100.00%
		Zhang's method	1117	1013	88.56%	100.00%	100.00%
		DPC-NADPIN	6464	6439	98.88%	*99.94*%	100.00%
		TS-OCD	1175	1157	97.26%	100.00%	100.00%
		DCA	1261	1261	100.00%	100.00%	100.00%
	BioGrid	ICJointLE-DPN	4896	4559	90.69%	100.00%	100.00%
		Zhang's method	1074	974	88.73%	100.00%	100.00%
		DPC-NADPIN	5437	5420	99.16%	100.00%	100.00%
		TS-OCD	1784	1749	96.56%	100.00%	100.00%
		DCA	1201	1199	98.45%	100.00%	100.00%
	DIP	ICJointLE-DPN	4398	4118	91.42%	100.00%	100.00%
		Zhang's method	836	725	85.18%	100.00%	100.00%
		DPC-NADPIN	3019	3007	99.24%	100.00%	100.00%
		TS-OCD	439	434	98.50%	100.00%	100.00%
		DCA	595	595	100.00%	100.00%	100.00%

GSE4987	STRING	ICJointLE-DPN	12283	11913	95.34%	*99.97*%	100.00%
		Zhang's method	1863	1712	89.36%	100.00%	100.00%
		DPC-NADPIN	4509	4498	99.45%	100.00%	100.00%
		TS-OCD	2302	2266	97.26%	100.00%	100.00%
		DCA	3212	3204	98.84%	*99.94*%	100.00%
	BioGrid	ICJointLE-DPN	10558	10100	93.25%	100.00%	100.00%
		Zhang's method	1821	1660	88.75%	100.00%	100.00%
		DPC-NADPIN	3657	3641	99.15%	100.00%	100.00%
		TS-OCD	3354	3295	97.12%	100.00%	100.00%
		DCA	2686	2678	98.46%	*99.60*%	100.00%
	DIP	ICJointLE-DPN	8745	8359	92.86%	100.00%	100.00%
		Zhang's method	1343	1177	85.76%	100.00%	100.00%
		DPC-NADPIN	6464	1668	99.66%	100.00%	100.00%
		TS-OCD	734	721	97.68%	100.00%	100.00%
		DCA	1269	1266	99.27%	100.00%	100.00%

## Data Availability

Algorithm ICJointLE-DPN is implemented in C++. The software suite of our method and the results produced by ICJointLE-DPN from three yeast PPI data sets STRING, BioGrid, and DIP are available at https://dx.doi.org/10.6084/m9.figshare.7824233. Or please contact to zhangjx@gxu.edu.cn.

## Supplementary Materials

Supplementary Materials For complexes identified by ICJointLE-DPN from different TI-PINs, their raw data of BP enrichment analyses and their results of significant statistics are, respectively, compressed in the following three packages. They are also available at https://dx.doi.org/10.6084/m9.figshare.7824233. Additional File 1: BioGrid_BP.rar for the protein complexes identified from TI-PINs constructed by integrating GSE3431 and GSE4987 into BioGrid respectively. Additional File 2: DIP_BP.rar for the protein complexes identified from TI-PINs constructed by integrating GSE3431 and GSE4987 into DIP, respectively. Additional File 3: STRING_BP.rar for the protein complexes identified from TI-PINs constructed by integrating GSE3431 and GSE4987 into STRING, respectively.
